# Comparing augmented reality-assisted and freehand external ventricular drain placement: a multicenter randomized controlled crossover phantom study

**DOI:** 10.1007/s00701-025-06738-7

**Published:** 2025-12-24

**Authors:** Jesse A. M. van Doormaal, Elisa Colombo, Jasper M. van der Zee, Wouter D. Maathuis, Maarten Bot, Patrick O’Donnell, Bachtiar Burhani, Luca Regli, Pierre A. J. T. Robe, Eelco W. Hoving, Tristan P. C. van Doormaal

**Affiliations:** 1 Department of Neurosurgery, University Medical Center Utrecht, Utrecht, The Netherlands; 2 Department of Neurosurgery, University Hospital Zürich, Zurich, Switzerland; 3 Department of Surgical Oncology, Princess Máxima Center for Pediatric Oncology, Utrecht, The Netherlands; 4 Department of Neurosurgery, Academic Medical Center Amsterdam, Amsterdam, The Netherlands; 5 Department of Neurosurgery, St. Elisabeth Hospital, Tilburg, The Netherlands; 6 Department of Neuro-Oncology, Princess Máxima Center for Pediatric Oncology, Utrecht, The Netherlands

**Keywords:** Augmented reality, External ventricular drain, Image-guided neurosurgery

## Abstract

**Background:**

External ventricular drain (EVD) placement is a common neurosurgical procedure with high rates of misplacement when performed using the freehand technique. With augmented reality (AR), the accuracy of EVD placement could be improved by providing a 3-D overlay, guiding optimal placement using a virtual trajectory superimposed over the patient. In this study, we aimed to assess the efficacy and usability of an AR application for assisting EVD placements which supported trajectory planning, point-based image-to-patient registration and 3-D stereoscopic projection.

**Method:**

We conducted a randomized controlled crossover trial involving 15 neurosurgical residents and one neurosurgeon, who performed 236 EVD procedures (118 AR-assisted and 118 freehand) on biomimetic phantoms. EVD placement accuracy was evaluated using the Kakarla scale, distance-to-target, angular inaccuracy, and depth inaccuracy. The total procedural time was recorded. The user experience was evaluated using the NASA Task Load Index (NASA-TLX) and the Usefulness, Satisfaction, and Ease of Use (USE) questionnaire.

**Results:**

AR-assisted placement achieved significantly higher rates of optimal placement (Kakarla grade 1: 57.6% vs 37.3%; p < .001), lower rates of erroneous placement (Kakarla grade 3: 21.2% vs 40.7%; p < .001), a lower distance-to-target (median, 7.2 mm vs 11.4 mm; p < .001) and lower angular inaccuracy (median, 5.58° vs 7.60°; p < .001). Procedural time was longer for AR (median, 7 min 30 s vs 1 min 11 s; p < .001). Participants rated the AR system favorably on the USE for ease of learning (mean, 6.09/7 [SD, 0.94]) and satisfaction (mean, 6.45/7 [SD, 0.69]), while NASA-TLX scores indicated similar workloads between AR and freehand techniques.

**Conclusions:**

AR improves the accuracy of EVD placement compared to the freehand technique, which is expected to improve the efficacy in clinical settings. It increases total procedural time but remains within clinically acceptable limits and provides favorable usability.

**Supplementary Information:**

The online version contains supplementary material available at 10.1007/s00701-025-06738-7.

## Introduction

External Ventricular Drain (EVD) placement is a common neurosurgical procedure generally performed in urgent cases of obstructive hydrocephalus (resulting from intraventricular/subarachnoid hemorrhage, meningitis, brain tumors, or ventriculoperitoneal shunt failure) and traumatic brain injury. Annually, more than 20,000 patients in the United States alone undergo this procedure [29].

Typically, EVD insertion is performed using the freehand technique, relying on anatomical landmarks for guidance [7]. Surgeons must mentally extrapolate an ideal EVD trajectory from their anatomical knowledge and 2-D images. However, this approach lacks consideration for anatomical variations and underlying pathologies that contribute to suboptimal placement [17]. The current clinical practice exhibits a high rate of misplacement, with suboptimal positioning or misplacement observed in approximately 26% to 28% of cases on post-operative imaging [31, 37]. Malposition of the EVD leads to an increased risk of revision/reinsertion [40], with reported freehand revision rates in literature ranging from 2.2% to 33.9% [12, 21, 24, 33, 36]. Multiple catheter passes through brain tissue before successful ventricle cannulation increase the risk of hemorrhage [27], which currently occurs in 7% of patients [31], and are also expected to heighten the risk of infection, which affects approximately 5% of patients [31].

Neuronavigation systems decrease the risk of suboptimal EVD placement below 10% [37]. However, these are costly devices that can be complex to set up and are often reliant on a skull clamp, which limits their use in emergencies or resource-constrained settings. Furthermore, neuronavigation systems use 2-D imaging on external screens, which forces a surgeon to divide attention between the screen and the patient while mentally translating the images to the 3-D anatomy.

3-D imaging and Augmented Reality (AR) technology could significantly improve the procedure, as it provides direct superimposition of stereoscopic 3-D imaging over the patient. State-of-the-art AR Head-Mounted Displays (AR-HMDs) facilitate marker tracking and spatial mapping using environment-recognizing sensors, which allows flexible image-to-patient registration and environmental tracking without large external sensors. This provides enhanced visualization of the ideal trajectory and internal anatomy [1, 28, 32, 38] and obviates the need to shift the surgeon’s attention to external monitors [5, 20]. Modern AR-HMDs are portable devices with a cost under $5000 [15].

Multiple AR applications have been developed for neurosurgery [4]. Prototype systems for AR-HMD assisted EVD placement demonstrate potential for improving catheter insertion accuracy [2, 3, 8, 13, 14, 16, 22, 34, 35, 41, 44, 46]. Nonetheless, neuronavigation using AR-HMDs has yet to gain widespread clinical adoption in neurosurgery, and only a limited number of studies have conducted large-scale controlled preclinical testing [2, 3, 14, 22, 35, 44, 46], generally without randomization of blinded outcome assessment. The main challenges of AR include shortcomings in segmentation, inaccurate image-to-patient registration, registration methods that are impractical in clinical settings and visual obstruction of the surgical field [5]. Furthermore, prototypical systems are not integrated into the hospital's digital infrastructure, hindering clinical implementation.

In this study, we investigate the AR-HMD system “Lumi”, which was designed to overcome these challenges. Lumi is intended to assist EVD placement while running locally on state-of-the-art AR-HMDs, without reliance on external sensors or computational units. Lumi includes trajectory planning, point-based image-to-patient registration with custom universally-fitting instruments, stereoscopic projection, and continuous optical tracking. Furthermore, the system is integrated with a previously described cloud-based data storage platform and automated segmentation system [6, 11, 42] which can be directly integrated in a hospital’s existing Picture Archiving and Communication System (PACS) infrastructure, establishing a comprehensive pipeline directly suitable for daily clinical workflows.

We aim to assess the system’s clinical efficacy by conducting a simulated randomized controlled trial with crossover of participants, comparing AR-assisted EVD placement to the conventional freehand technique. This trial was carried out by neurosurgeons and neurosurgical residents in a simulated operating room, utilizing anatomically realistic phantoms to replicate challenging real-world conditions. Clinical efficacy was evaluated using blinded assessment of placement accuracy, and by measuring the procedural time. User experience was assessed using validated questionnaires.

## Methods and materials

### Participants

16 participants were recruited from the participating study centers, comprising 1 neurosurgeon and 15 neurosurgical residents from UMC Utrecht, ETZ Tilburg, Amsterdam UMC, and University Hospital Zürich. Participants were selected from a diverse range of clinical experience levels. Six enrolled participants were ANIOS (non-specialist residents), nine were AIOS (residents in specialist training), and one was a neurosurgeon. Based on self-reported experience, four participants had placed fewer than 10 EVDs in their career, five had placed between 10 and 25, two between 25 and 50, and five had placed more than 50. Characteristics of the participants are presented in Table 1.

**Table 1:** Base characteristics of participants

Characteristic	Percentage % of participants (*N*)
Total (No.)	16
Study center	
University Medical Center Utrecht	43.8 (7)
Elisabeth-Tweesteden Hospital	12.5 (2)
Amsterdam UMC	18.8 (3)
University Hospital Zürich	25 (4)
Level of medical training	
ANIOS^*^	37.5 (6)
AIOS^**^	56.3 (9)
Neurosurgeon	6.3 (1)
Self-estimated amount of placed EVDs in career	
< 10	25.0 (4)
10–25	31.3 (5)
25–50	12.5 (2)
> 50	31.3 (5)
AR experience	
None	18.8 (3)
Basic***	62.5 (10)
Experienced****	18.8 (3)

*ANIOS is a Dutch clinical level, defined as a physician who is not currently enrolled in medical speciality training

**AIOS is a Dutch clinical level, defined as a physician who is currently enrolled in medical specialty training

***”Basic” AR experience was defined as using AR at least twice in clinical settings

****”Experienced” was defined as using AR at least monthly in clinical settings, for at least 6 months

### Phantoms

To simulate EVD procedures, six biomimetic 3-D printed head phantoms were fabricated, based on a previously described phantom for EVD placement which has been validated for its realistic tactile fidelity and biomimetic materials [43]. These phantoms were based on six CT scans selected from an imaging database of patients with subarachnoid hemorrhage [39]. Written general consent for further use of their patient-specific data was previously obtained from all patients or their legal representatives [39]. To simulate scenarios with an expected high benefit of AR, challenging cases with small or shifted ventricles were selected based on radiological characteristics (Table 2). The procedural difficulty of the phantoms was assigned as either “moderate”, “difficult” or “very difficult”. To guarantee high segmentation accuracy, 3-D models of the skin, skull, brain and ventricles were manually segmented within a medical image analysis platform (3D Slicer, Harvard University, Cambridge, USA) [10, 18]. Within the CT, a neurosurgeon indicated optimal bilateral target points in the frontal horn.

**Table 2:** Anatomical characteristics of CTs used for designing the phantoms

Phantom Type	Pathology	Ventricular Volume (cc)	Evans Index	Bicaudate Index	Midline shift	Intraventricular blood	Difficulty
1	Healthy	22.44	0.232	0.142	0	N	Moderate
2	Healthy	40.91	0.288	0.123	0	N	Moderate
3	Subarachnoid hemorrhage	8.04	0.297	0.186	6.1	Y	Difficult
4	Subarachnoid hemorrhage	20.86	0.242	0.108	0	Y	Difficult
5	Subarachnoid hemorrhage	6.58	0.105	0.093	2.7	N	Very difficult
6	Subarachnoid hemorrhage	11.22	0.184	0.066	8.5	N	Very difficult

**Fig. 1:**
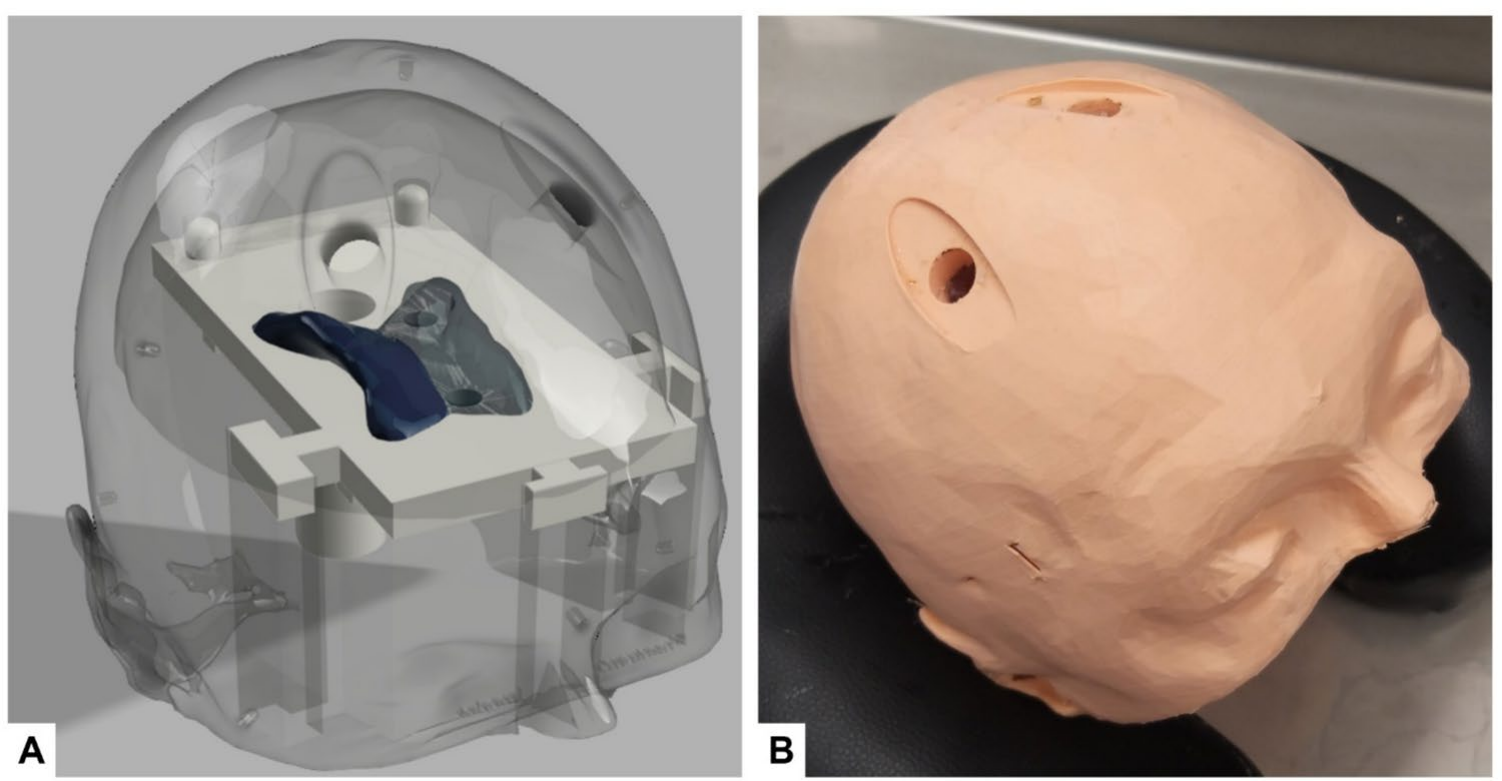
**a** Internal structure of the 3-D printed phantom, including ventricular cavities. **b** External appearance of the phantom with burr holes at Kocher's point

Based on the segmentations, anatomical phantoms were 3-D printed in polylactic acid (Ender 3 V3 SE, Shenzhen Creality 3D Technology Co., Ltd., Shenzhen, China) and filled with a 1.0% agarose polysaccharide gel to simulate brain tissue (Fig. 1). The skin layer included pre-printed bilateral ‘incisions’ and 13 mm burr holes located 10 cm posterior to the nasion and 3 cm lateral to the midline [7]. Air-filled ventricular cavities were incorporated in their original anatomical positions using an imprint of the ventricles. The phantoms featured a removable bottom section, allowing inspection of the drain position.

### AR device

The investigational AR-HMD application ‘Lumi’ was iterated on a previously described AR platform which facilitates cloud storage, automatic segmentation and visualization features for MRI and CT images [6, 11, 42]. Lumi offers EVD trajectory planning, point-based image-to-patient registration and EVD guidance. Lumi was developed using the real-time development platform Unity (Unity Technologies, San Francisco, USA) in C#. Lumi was operated using state-of-the-art AR-HMDs (Hololens 2, Microsoft, Redmond, USA).

### Experiment training

Prior to the experiments, participants completed a standardized two-hour training session. This training covered technique both freehand and AR-assisted methods for placing an EVD. The freehand training section focused on the fundamental techiques of placing a freehand EVD and included an explanation of measuring distances and angles to the optimal target point on the CT. The content was tailored to the participant’s experience level. The AR training section involved a demonstration and at least three practice rounds on a training phantom. Participants could only proceed to the experimental phase after demonstrating independent proficiency in both modalities.

### Trial design

To assess the efficacy of Lumi, we conducted a ‘simulated’ randomized controlled trial with crossover of participants. Participants conducted EVD placements on a randomly selected subset of three or six phantoms, allowing flexibility to accommodate differences in the participant’s schedule and procedural speed. To ensure balanced exposure to varying difficulty levels, each participant was assigned either one or two phantoms from each of the “moderate,” “difficult,” and “very difficult” categories.

For every phantom, participants performed bilateral placements using the AR approach and bilateral placements using the freehand approach, ensuring a balanced level of placement difficulty between the two methods. Consequently, the total number of placements per participant ranged from 12 to 24.

Participants were randomized to perform the initial bilateral placements on the first phantom using either the AR or freehand method, followed by bilateral placements using the alternative method. The order of techniques was alternated for each subsequent phantom. To ensure balance between groups, randomization was stratified based on the phantom subtype.

### AR-assisted EVD placement

**Fig. 2:**
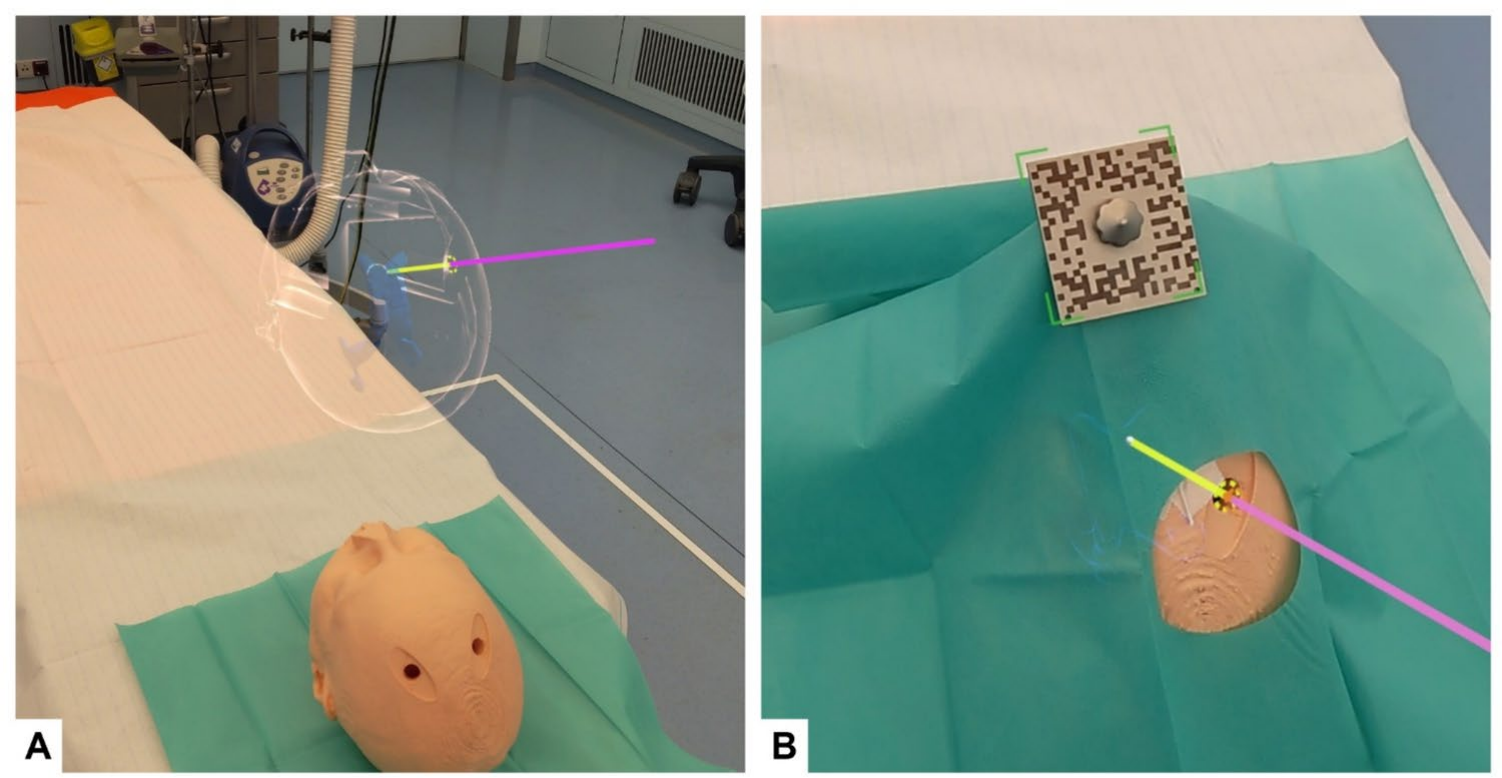
**a** Trajectory planning using a beige, glass-shaded skin model and transparent blue ventricles. The planned trajectory is an opaque 3-D line, with the extracranial segment in purple and the intracranial segment in yellow. **b** AR overlay during EVD placement. The reference device’s optical marker is outlined in green over a sterile sheet. Ventricles are rendered as a blue, glass-shaded 3-D model, with the same color-coded trajectory and a white sphere marking the target

Prior to the experiment, 3-D models of the phantom, skull, brain and ventricles were uploaded to Lumi. Within Lumi, the primary researcher planned six anatomical landmarks (tragus left/right, lateral canthus left/right and medial canthus left/right) and the EVD trajectory (Fig. 2).

Before each procedure, participants were provided access to the patient’s original CT scan and pre-defined optimal target point within 3D Slicer. Participants were allowed to indicate Kocher’s point and measure the required EVD depth using measuring functions.

**Fig. 3:**
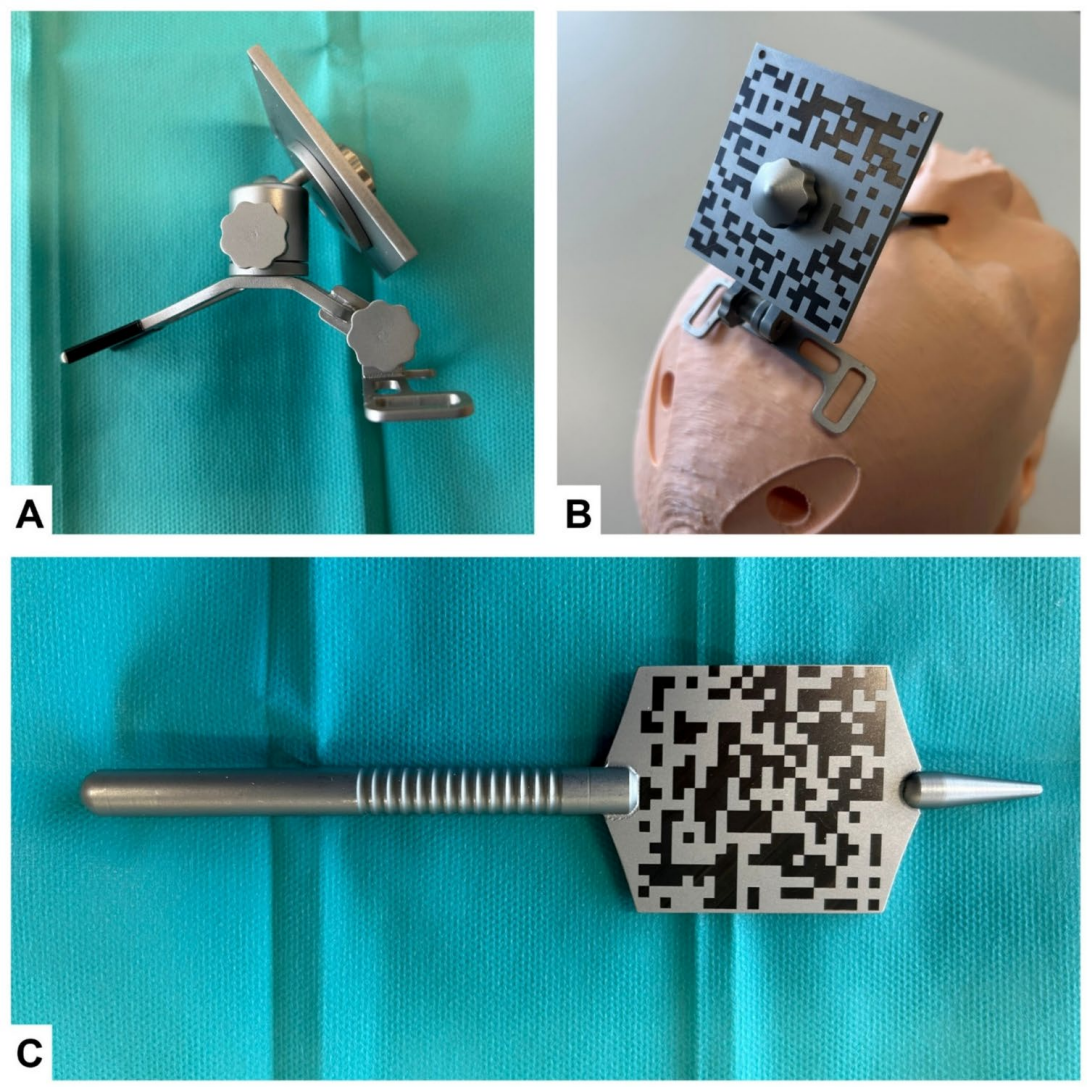
**a** Reference device with removable optical marker, side view. **b** Reference device fitted on phantom. **c** Probe equipped with an optical marker for point-based registration

During the procedure, participants first positioned the reference device. This stainless-steel universal patient mount (Fig. 3A) is adjustable via a ball joint and a hinge joint, and is designed to be positioned on the patient's nose and forehead (Fig. 3B). It features a removable, interchangeable 50 × 50 mm laser-engraved optical marker that enables continuous tracking during both image-to-patient registration and the subsequent procedure. To compensate for any optical projection error, a visual calibration correction can be applied to align the AR overlay with the optical marker.

Subsequently, image-to-patient registration was performed. Hereto, the participants indicated the previously planned anatomical landmarks on the real patient using a gaze-activated stainless-steel probe featuring a 50 × 50 mm laser-engraved optical marker (Fig. 3C). Following registration, the participants verified the accuracy of the process by assessing the superimposed skin outline and the Fiducial Registration Error (FRE) [25, 26], which was projected within Lumi. After verification, participants could optimize the projected virtual trajectory by adjusting the trajectory endpoint to ensure alignment with the burr hole.

The participants then draped the patient with a sterile sheet. During this process, the non-sterile optical marker on the reference device was replaced with a sterile marker, which was affixed atop the drapes in sterile fashion. Next, the virtual EVD trajectory, the ventricles and the target point were now projected onto the patient (Fig. 2). The distance between the skull surface and the target point was projected to aid participants in determining the required depth for EVD insertion. Assisted by the AR overlay, participants attempted EVD placement in the ipsilateral frontal horn, aiming to position the catheter as close as possible to the planned target point. Standard practices, such as palpating anatomical landmarks or applying reference stickers, were permitted. The procedural time, which included point-based registration, draping and EVD placement, was recorded.

The full workflow of the AR application is demonstrated in Online Resource 1.

### Freehand EVD placement

For freehand EVD placements, participants first accessed the patient's original CT scan, which the phantom was modeled after, via a medical imaging platform (3D Slicer, Harvard University, Cambridge, USA). An optimal pre-planned target point was displayed within the CT. Participants were encouraged to take measurements using the ruler and curve functions and to calculate compensation for any midline shift of the ventricles.

During the procedure, participants were instructed to drape the phantom using a sterile sheet. Next, the participants attempted EVD placement in the ipsilateral frontal horn, aiming the catheter as close as possible to the optimal target point on the CT. Participants were encouraged to use the same supportive tools and techniques they would employ in clinical practice to guide optimal placement, such as adhesive markers, skin markers, and palpation of anatomical landmarks. The procedural time, which included draping and EVD placement, was recorded.

### Measurements

After EVD placement, the bottom of the phantom was removed to expose the ventricular cavity and the drain tip’s position was documented through high resolution photographs. For a subset of procedures, an Electromagnetic (EM) tracking system (Aurora, Northern Digital Incorporated, Waterloo, Canada) with submillimeter accuracy [19], registered to the phantom's position, was employed to determine the drain tip's location relative to the target point.

Following the experiment, two blinded researchers independently assessed the Kakarla grade for each placement using the photographs. The Kakarla grade evaluates EVD placement accuracy, with placement in the ipsilateral frontal horn classified as optimal (grade 1), placement in the contralateral ventricle or posterior to the foramen of Monro as suboptimal (grade 2), and placement outside the ventricles as erroneous (grade 3) [17]. Discrepancies were resolved through panel discussion to reach a consensus.

Using the EM measurements, a Python script was employed to calculate several metrics:

1. The Euclidean distance between the EVD tips and their optimal targets.
2. The angular deviation between the vector from Kocher’s point to the EVD tip and the vector from Kocher’s point to the optimal target.
3. The depth inaccuracy, defined as the length difference between these two vectors.

To evaluate user experience after completion of all experiments, participants completed the National Aeronautics and Space Administration Task Load Index (NASA-TLX) [30] for both the freehand and AR tasks, assessing subjective workload across the mental, physical, temporal, performance, effort and frustration domains. Additionally, the Usefulness, Satisfaction, and Ease of Use (USE) [23] questionnaire was administered for the AR task, which evaluates the subjectively experienced effectiveness and user-friendliness.

### Statistical analysis

A significance level of *p* < 0.05 was used for all statistical tests. All statistical tests and graphical representations were performed in Python using the pandas, Numpy and Scipy libraries. Prior to analysis, a Shapiro–Wilk test and histogram inspection were conducted to assess the normality of data distributions. A Stuart-Maxwell test was used to compare the Kakarla distributions between modalities (AR and freehand). Ordinal logistic regression was employed to model the effects of modality and clinical experience (> 25 vs. < 25 EVD placements) on the Kakarla grade. A Wilcoxon signed-rank Test was used to compare the distance-to-target, angular inaccuracy, depth inaccuracy and NASA-TLX subscale scores between modalities.

## Results

Each participant successfully completed the training and performed between 12 and 24 EVD placements. This resulted in a total of 236 paired procedures, with 118 performed using the AR technique and 118 using the freehand technique. The experiment results have been summarized in Table 3.

**Table 3:** Overview of results

Metric	AR Technique		Freehand Technique		*p*-value
Kakarla Grade	Proportion	95% CI	Proportion	95% CI	
Grade 1 (%)	57.6	48.7–66.5	37.3	28.6–46.0	< *0.001*
Grade 2 (%)	21.2	13.8–28.6	22.0	14.6–29.5	< *0.001*
Grade 3 (%)	21.2	13.8–28.6	40.7	31.8–49.5	< *0.001*
Trajectory Analysis	**Median**	**IQR**	**Median**	**IQR**	
Distance-to-target (mm)	7.21	5.22	11.40	8.76	< *0.001*
Angular inaccuracy (°)	5.58	4.18	7.60	8.85	< *0.001*
Depth inaccuracy (mm)	2.86	2.67	3.23	4.73	*0.030*
Procedural Time	**Median**	**IQR**	**Median**	**IQR**	
Time (min:s)	7:30	3:33	1:11	0:37	> *0.500*
NASA-TLX	**Mean**	**SD**	**Mean**	**SD**	
Mental	11.30	4.66	10.93	5.05	> *0.500*
Physical	8.63	4.99	6.77	4.14	*0.016*
Temporal	8.77	4.17	5.77	3.14	*0.021*
Performance	7.97	5.11	9.70	4.27	*0.313*
Effort	10.10	4.01	8.77	4.34	*0.095*
Frustration	6.83	4.24	8.50	4.89	*0.221*

### Placement accuracy

**Fig. 4:**
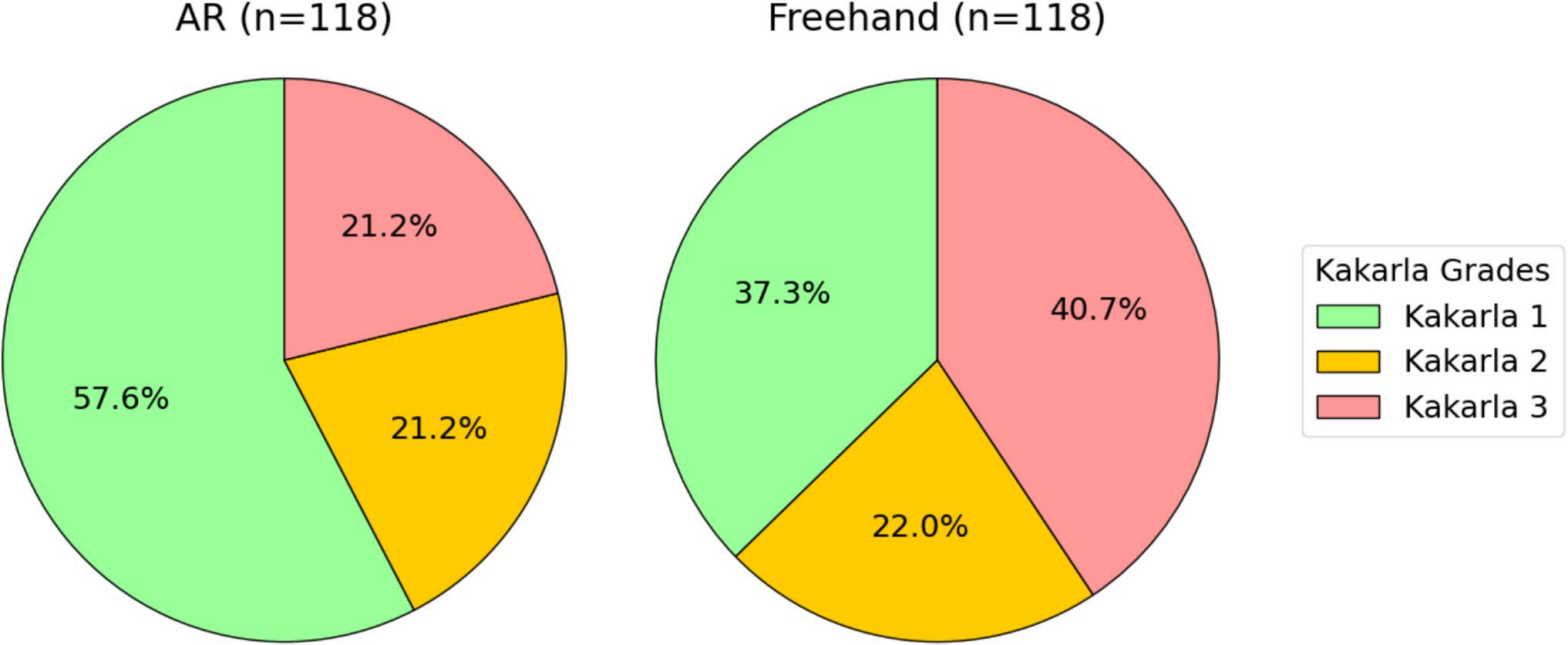
Distribution of Kakarla grades in the AR and freehand groups

The AR group achieved a higher proportion of optimal Kakarla grade 1 placements (57.6% [95% CI, 48.7%−66.5%]) compared with the freehand group (37.3% [95% CI, 28.6%−46.0%]). The proportion of suboptimal grade 2 placements was similar between groups (AR: 21.2% [95% CI, 13.8%−28.6%] vs freehand: 22.0% [95% CI, 14.6%−29.5%]), while erroneous grade 3 misplacements were less frequent in the AR group (21.2% [95% CI, 13.8%−28.6%]) compared with the freehand group (40.7% [95% CI, 31.8%−49.5%]) (Fig. 4). This difference in distributions was statistically significant (χ^2^(2) = 55.04; *p* < 0.001). The relative risk of misplacement (Kakarla 3) was 0.52 when using AR.

**Fig. 5:**
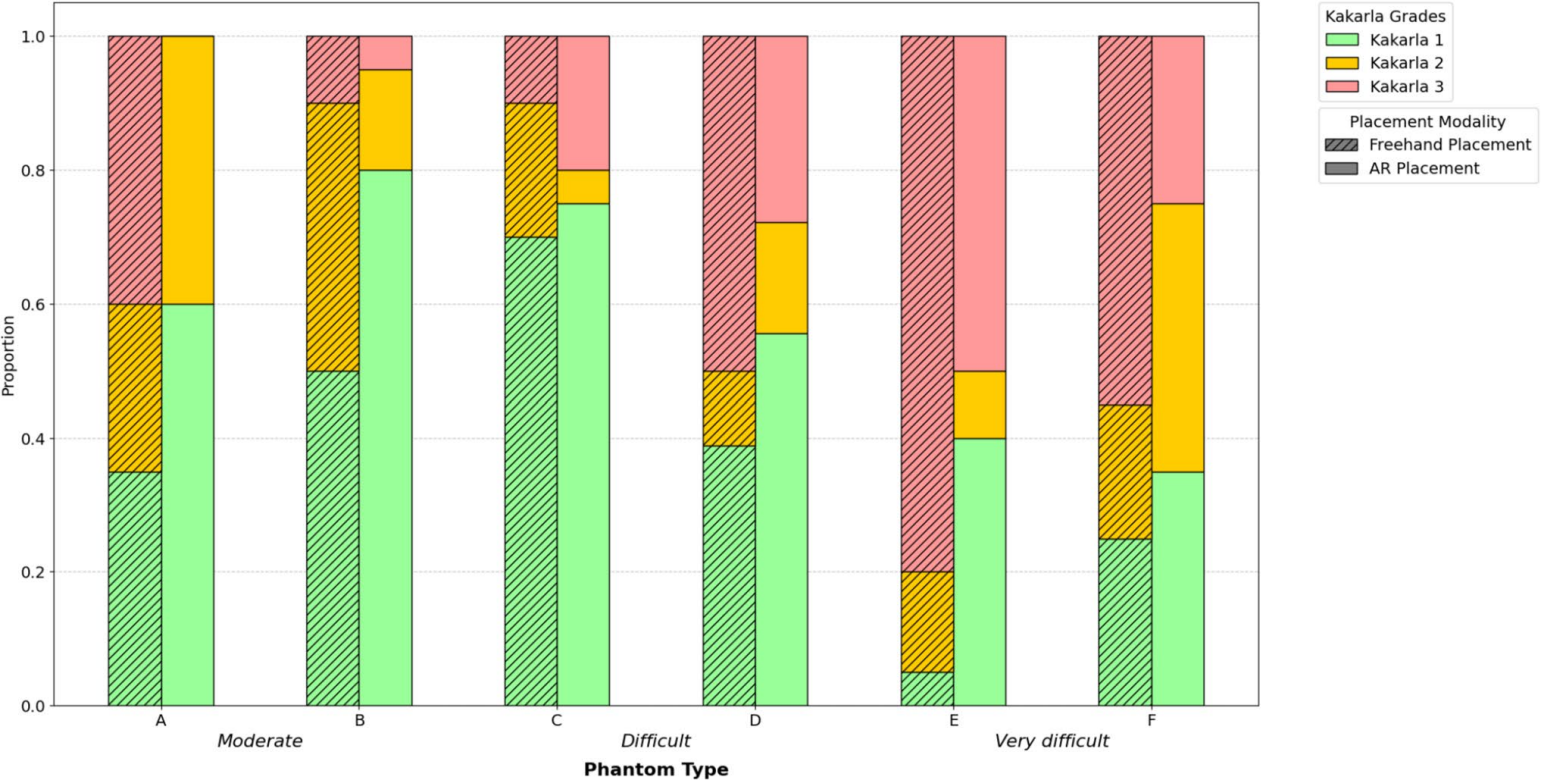
Kakarla scores for each phantom subtype

Ordinal logistic regression revealed that AR was associated with lower odds of a worse Kakarla grade (coefficient −0.87; OR, 0.42, *p* < 0.001) compared with freehand, whereas clinical experience (> 25 vs. < 25 EVD placements in career) did not significantly influence the Kakarla grade (coefficient −0.09; OR, 0.92; *p* = 0.73). Subgroup analysis showed lower Kakarla scores for the AR modality for all phantom subtypes across all difficulty categories (Fig. 5).

**Fig. 6:**
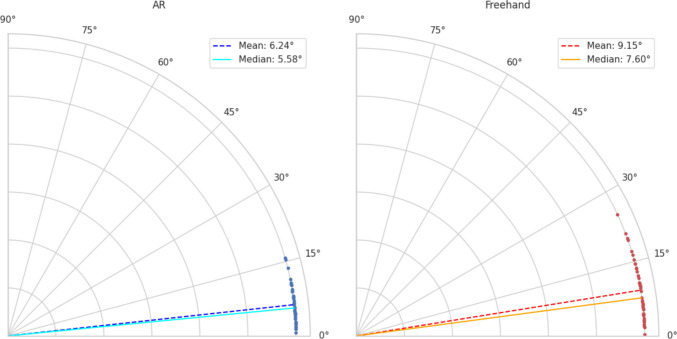
Comparison of angular inaccuracy between AR-assisted and freehand EVD placement

Geometric analysis using EM measurements was conducted for a subset of 43 AR placements and 43 freehand placements. The AR group demonstrated a smaller distance-to-target (median, 7.2 mm [IQR, 5.2 mm]) compared to the freehand group (median, 11.4 mm [IQR, 8.8 mm]), which was statistically significant (*W* = 174.0; *p* < 0.001). The AR group had a lower angular inaccuracy (median, 5.58° [IQR, 4.18°]) compared with the freehand group (median, 7.60° [IQR, 8.85°]) (Fig. 6), which was statistically significant (*W* = 17.0; *p* < 0.001). The AR group had a smaller depth inaccuracy (median, 2.9 mm [IQR, 2.7 mm]) compared to the freehand group (median, 3.2 mm [IQR, 4.7 mm]), which was also statistically significant (*W* = 294.0; *p* = 0.030). The mean FRE of the AR registrations was 3.7 mm (SD, 0.6 mm).

### Procedural time

The AR group exhibited a longer procedural time (median, 7 min 30 s [IQR, 3 min 33 s]) compared to the freehand group (median, 1 min 11 s [IQR, 37 s]), suggesting a median extension of 6 min and 19 s to the intraoperative workflow.

### User experience

**Fig. 7:**
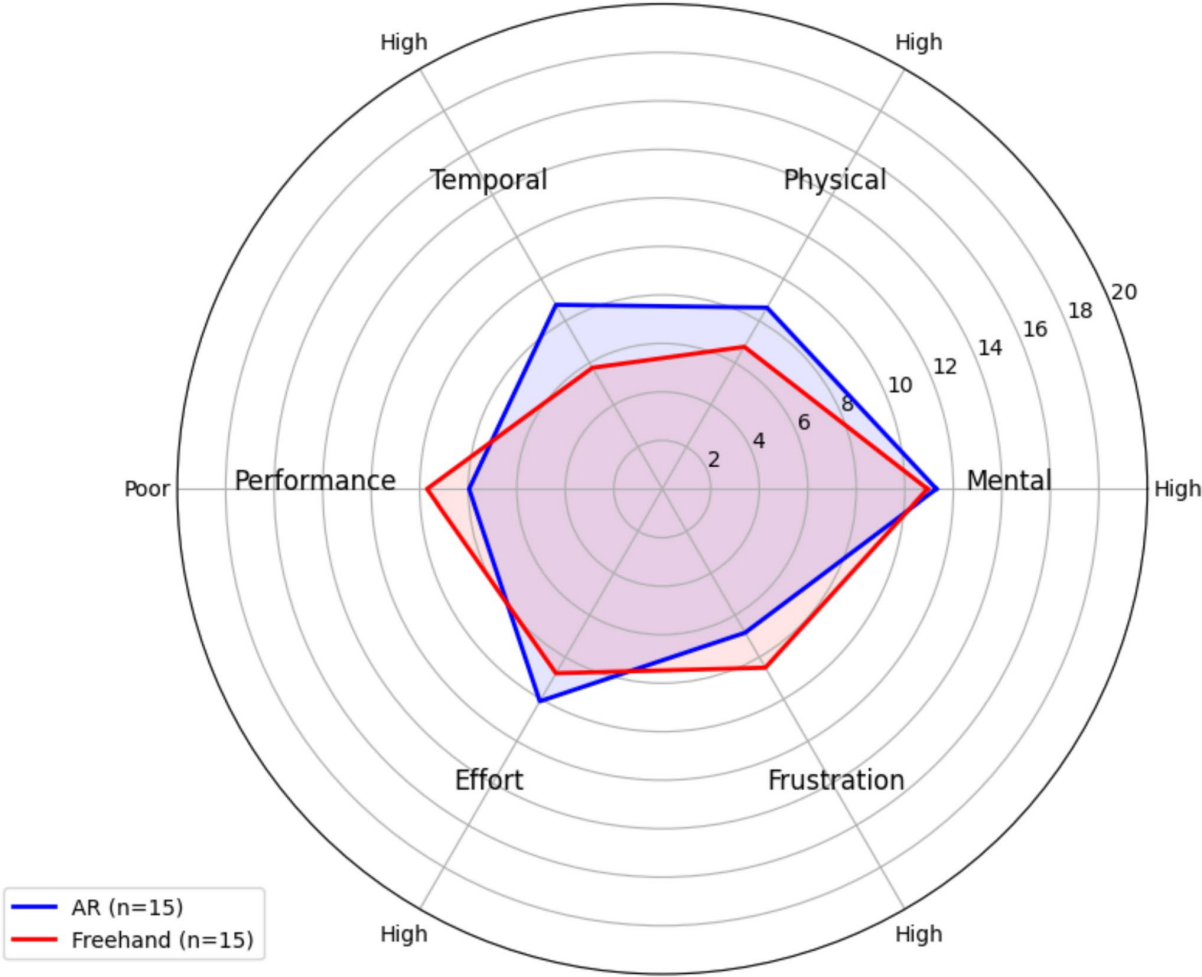
NASA-TLX workload scores for AR-assisted and freehand EVD placement

The NASA-TLX scale was completed by 15 of 16 participants. The total workload score for the AR modality was somewhat high* (*mean*,* 51.36 [SD, 18.87]), though similar to the freehand scores *(*mean*,* 48.54 [SD, 20.29]) (Fig. 7). There was no statistically significant difference in total workload scores between the AR and Freehand modalities (W = 22.50, *p* = 0.108). However, significantly higher scores were observed for the AR modality in the physical demand (W = 6.00, *p* = 0.016) and temporal demand (W = 12.50, *p* = 0.021) subscales.

**Fig. 8:**
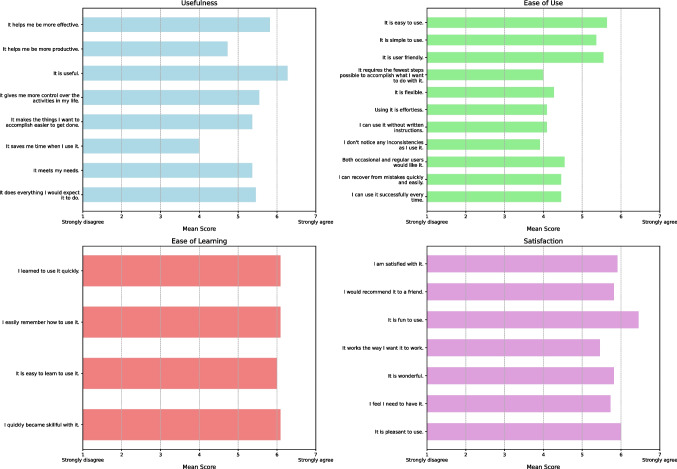
USE questionnaire scores

The USE questionnaire was completed by 15 of 16 participants (Fig. 8). In the"Usefulness"subscale, participants rated the system as very useful (mean, 6.27 [SD, 0.65]) and capable of improving the user’s effectiveness (mean, 5.82 [SD, 0.87]), though scores were lower for saving time (mean, 4.00 [SD, 1.61]). In the"Ease of Use"subscale, participants found the system easy to use (mean, 5.64 [SD, 1.12]) and user-friendly (mean, 5.55 [SD, 0.93]). Lower ratings were given for requiring the fewest steps possible (mean, 4.00 [SD, 1.67]) and being effortless to use (mean, 4.09 [SD, 1.38]). In the"Ease of Learning"subscale, participants rated the system as easy to learn (mean, 6.09 [SD, 0.94]) and easy to remember how to operate (mean, 6.09 [SD, 0.83]). Finally, in the"Satisfaction"subscale, participants rated the system highly for being fun to use (mean, 6.45 [SD, 0.69]) and pleasant overall (mean, 6.00 [SD, 0.77]).

## Discussion

### Summary

In this phantom study, we developed an AR system for assisting EVD placement, which is readily integrable into existing digital hospital infrastructure and clinical workflows. We assessed the system’s efficacy by measuring placement accuracy, procedural time and user experience in a series of realistically simulated EVD procedures. Our findings indicate that AR improved the accuracy of EVD placements compared to the freehand technique, with favorable Kakarla grades and a reduced distance-to-target, likely due to enhanced angular and depth accuracy. The intraoperative procedural time was extended by less than 7 min when using AR. Users reported that the system was quick to learn, easy to use, and effective as a surgical tool, while it did not result in a higher perceived workload.

This study confirmed that superimposing 3-D models onto the surgical field using AR enhances the understanding of surgical anatomy, thereby improving surgical outcomes compared to the freehand approach. AR increased the procedural time due to the additional required steps, although we consider it within clinically acceptable limits. Additionally, user questionnaires and procedural time indicated that the AR system is simple and quick to set up and operate, providing an effective alternative to conventional neuronavigation, particularly in emergency or low resource settings.

### Literature context

Several AR-HMD applications for assisting EVD placements have been described, generally reporting favorable outcomes and user experiences [2, 3, 8, 13, 14, 16, 22, 34, 35, 44]. However, many studies rely on image-to-patient registration using a pre-determined rigid relationship between a non-sterile marker and a phantom, which is not feasible in clinical settings [3, 8, 34, 35]. Other studies rely on external tracking devices, which results in a complex setup and requires more physical space, making it impractical for emergency procedures [2, 8]. Our AR system addresses this limitation by utilizing time-efficient point-based registration with a universally fitting reference device designed for sterile environments, based on optical tracking using the AR-HMD’s visual light camera, enabling assisted EVD placement across various clinical scenarios. Furthermore, none of the studies offer a complete clinical workflow, with image retrieval and segmentation integrated into existing hospital infrastructure. To address this gap, our application is built as an extension on an existing AR platform with PACS integration and automatic CT segmentation.

Despite AR guidance, a certain degree of inaccuracy is inherently inevitable due to the compound error of imperfect image-to-patient registration and operator error when following the virtual trajectory with the EVD. Accuracy is most reliably compared by evaluating the distance-to-target, as this metric remains unaffected by variations in ventricle sizes or phantom designs. Regarding distance-to-target, our system is generally comparable to or more accurate than similar systems described in the literature [3, 8, 14, 35, 44]. While a subset of studies describe higher accuracy [2, 13, 22], these rely on either a pre-determined rigid marker-to-patient relationship or advanced tracking methods requiring external hardware, which limits their clinical applicability. Comparing the Kakarla grade is complex, as each study uses unique phantoms with varying complexity and operators with different clinical experience. In our study, four out of six phantoms were designed to have small ventricles to simulate difficult cases in which AR would have a relatively large benefit. The misplacement rate in this technically challenging population was favorable, exhibiting a Kakarla 3 rate of 21.2% and a relative risk of misplacement of 0.52 compared to freehand. The relative outcomes are consistent with a previous study comparing the Kakarla grades of AR versus freehand [44].

### Study strengths

A key strength of this study is its statistically robust design, incorporating a randomized crossover methodology with blinded outcome assessment—a novel approach not previously reported in literature, which minimizes the bias of assessment. Furthermore, all experiments were conducted in realistic simulated operating rooms using custom biomimicking phantoms derived directly from patient imaging data, which closely emulates clinical practice. Additionally, the Kakarla grade, the primary outcome measure of this study, is a clinically relevant metric that can be linked to the expected risk of harm in real patients. Lastly, the surgeon's user experience was assessed using validated questionnaires, providing insights into system usability and the perceived added value of the system.

### Study limitations

However, some study limitations persist. Four out of six phantoms were designed to simulate challenging cases, as reflected in the relatively low rate of optimal Kakarla 1 placements, despite using AR. Ventriculomegaly is normally defined by an Evans index of > 0.30 [9], as opposed to the average Evans index of 0.225 in our population. Additionally, two phantoms featured significant midline shift. Therefore, the absolute Kakarla rates reported in this study are a reflection of challenging cases, and cannot be directly translated to an average population of patients requiring an EVD, who typically have ventriculomegaly. Nonetheless, we expect the relative benefit of AR to translate even in non-complex cases, as AR-associated benefit was still observed in the two phantoms with the largest ventricles.

Moreover, the AR method was exclusively compared to the freehand approach, with no evaluation against conventional neuronavigation techniques. Consequently, the precise differences in accuracy, user experience, and procedural time between AR and conventional neuronavigation remain unclear. Lastly, the procedure encompassed only image-to-patient registration, draping, and EVD placement. Therefore, the measured procedural time and accuracy metrics were not influenced by trajectory and landmark planning on the hologram, Kocher’s point identification on the patient, incision, and trepanation. Although early testing indicates that these additional steps likely have a minimal impact on placement accuracy, the reported increase in procedural time reflects only the intraoperative phase.

### Study impact & future directions

This preclinical study is an essential prerequisite for the conduct of a clinical trial to evaluate the safety of AR-assisted EVD placement in clinical practice and to determine whether its efficacy, as observed in this study, extends to real-world settings. Furthermore, future research could explore the impact of AR on the learning curve of neurosurgical trainees.

In the future, we aim to decrease the procedural time by automizing planning steps, such as defining anatomical landmarks, EVD trajectory planning and image-to-patient registration using surface recognition. Furthermore, we aim to explore integrated infrared tracking using the AR-HMD’s built-in infrared camera [44, 45], which could enhance placement accuracy without increasing system complexity. Improving the system's precision could potentially extend its application to additional trajectory-based procedures, such as brain biopsies or deep brain stimulation. The integration and refinement of automatic segmentation, automatic planning, automatic image-to-patient registration, and surgical robotics may represent the next step toward fully automated and precise EVD insertion.

## Conclusion

This study demonstrates the efficacy and usability of our AR-HMD application for assisting EVD placement. The findings indicate that our AR system enhances clinical outcomes, including the Kakarla grade and geometric accuracy, with relevant margins. While the system increases procedural time, this remains within acceptable clinical limits. Furthermore, the results indicate that the software is adequately optimized for effective use by neurosurgical personnel, offering a positive user experience. This AR system holds significant promise as a tool for improving outcomes in patients requiring an EVD.

## Supplementary Information

Below is the link to the electronic supplementary material.

Supplementary Material 1 (MP4 283 MB)

## Data Availability

The database containing all anonymized data of this study was publicly published at the following URL: https://github.com/JessevanDo/PhantomStudyPublicRepo.

## Abbreviations

AR: Augmented Reality
AR-HMD: Augmented Reality Head-Mounted Display
CI: Confidence Interval
CT: Computed Tomography
EM: Electromagnetic
EVD: External Ventricular Drain
FRE: Fiducial Registration Error
IQR: Interquartile Range
MRI: Magnetic Resonance Imaging
NASA-TLX: NASA Task Load Index
OR: Operating Room
PACS: Picture Archiving and Communication System
SD: Standard Deviation
USE: Usefulness, Satisfaction and Ease of Use

## References

[1] Aguilar-Salinas P, Gutierrez-Aguirre SF, Avila MJ, Nakaji P (2022) Current status of augmented reality in cerebrovascular surgery: a systematic review. Neurosurg Rev. 10.1007/s10143-022-01733-3 35149900 10.1007/s10143-022-01733-3

[2] Benmahdjoub M, Thabit A, Van Veelen MLC, Niessen WJ, Wolvius EB, Walsum TV (2023) Evaluation of AR visualization approaches for catheter insertion into the ventricle cavity. IEEE Trans Vis Comput Graph. 10.1109/TVCG.2023.3247042 37027733 10.1109/TVCG.2023.3247042

[3] Bounajem MT, Cameron B, Sorensen K et al (2023) Improved accuracy and lowered learning curve of ventricular targeting using augmented reality—phantom and cadaveric model testing. Neurosurgery. 10.1227/neu.0000000000002293 36562619 10.1227/neu.0000000000002293

[4] Cannizzaro D, Zaed I, Safa A et al (2022) Augmented reality in neurosurgery, state of art and future projections. A systematic review. Front Surg 9:864792. 10.3389/fsurg.2022.864792 10.3389/fsurg.2022.864792 PMC8961734 35360432

[5] Cho J, Rahimpour S, Cutler A, Goodwin CR, Lad SP, Codd P (2020) Enhancing reality: a systematic review of augmented reality in neuronavigation and education. World Neurosurg. 10.1016/j.wneu.2020.04.043 32311561 10.1016/j.wneu.2020.04.043

[6] de Boer M, Kos TM, Fick T et al (2024) NnU-net versus mesh growing algorithm as a tool for the robust and timely segmentation of neurosurgical 3D images in contrast-enhanced T1 MRI scans. Acta Neurochir (Wien). 10.1007/s00701-024-05973-8 38376564 10.1007/s00701-024-05973-8 PMC10879314

[7] Dossani RH, Patra DP, Terrell DL, Willis B (2021) Placement of an external ventricular drain. N Engl J Med. 10.1056/NEJMvcm1805314 33497549 10.1056/NEJMvcm1805314

[8] Eom S, Kim S, Jackson J, Sykes D, Rahimpour S, Gorlatova M (2024) Augmented reality-based contextual guidance through surgical tool tracking in neurosurgery. IEEE Trans Vis Comput Graph. 10.1109/TVCG.2024.3390680 10.1109/TVCG.2024.3390680 38635386

[9] Evans WA Jr (1942) An encephalographic ratio for estimating ventricular enlargement and cerebral atrophy. Arch Neurol Psychiatry. 10.1001/archneurpsyc.1942.02290060069004

[10] Fedorov A, Beichel R, Kalpathy-Cramer J et al (2012) 3D slicer as an image computing platform for the quantitative imaging network. Magn Reson Imaging. 10.1016/j.mri.2012.05.001 22770690 10.1016/j.mri.2012.05.001 PMC3466397

[11] Fick T, van Doormaal JAM, Tosic L et al (2021) Fully automatic brain tumor segmentation for 3D evaluation in augmented reality. Neurosurg Focus 51(2):E14 10.3171/2021.5.FOCUS21200 10.3171/2021.5.FOCUS21200 34333477

[12] Foreman PM, Hendrix P, Griessenauer CJ, Schmalz PGR, Harrigan MR (2015) External ventricular drain placement in the intensive care unit versus operating room: evaluation of complications and accuracy. Clin Neurol Neurosurg. 10.1016/j.clineuro.2014.09.026 25436470 10.1016/j.clineuro.2014.09.026

[13] Gibby W, Cvetko S, Gibby A et al (2022) The application of augmented reality–based navigation for accurate target acquisition of deep brain sites: advances in neurosurgical guidance. J Neurosurg. 10.3171/2021.9.JNS21510 34920422 10.3171/2021.9.JNS21510

[14] Grunert R, Winkler D, Wach J et al (2024) Imaginer: improving accuracy with a mixed reality navigation system during placement of external ventricular drains. A feasibility study. Neurosurg Focus. 10.3171/2023.10.FOCUS23554 38163343 10.3171/2023.10.FOCUS23554

[15] HoloLens 2—Pricing and Options | Microsoft HoloLens. https://www.microsoft.com/en-us/hololens/buy. Accessed 27 Nov 2024

[16] Janssen A, Wang A, Dumont AS, Delashaw J (2024) Augmented reality-guided external ventricular drain placement: a case report. Cureus 16(7):e64403. 10.7759/cureus.64403 10.7759/cureus.64403 PMC11317059 39130984

[17] Kakarla UK, Kim LJ, Chang SW, Theodore N, Spetzler RF (2008) Safety and accuracy of bedside external ventricular drain placement. Neurosurgery. 10.1227/01.neu.0000335031.23521.d0 18728595 10.1227/01.neu.0000335031.23521.d0

[18] Kikinis R, Pieper SD, Vosburgh KG (2014) 3D slicer: a platform for subject-specific image analysis, visualization, and clinical support. Intraoperative Imaging Image-Guid Ther. 10.1007/978-1-4614-7657-3_19

[19] Koivukangas T, Katisko JP, Koivukangas JP (2013) Technical accuracy of optical and the electromagnetic tracking systems. Springerplus. 10.1186/2193-1801-2-90 10.1186/2193-1801-2-90 PMC3622743 23586003

[20] Lee C, Wong GKC (2019) Virtual reality and augmented reality in the management of intracranial tumors: a review. J Clin Neurosci. 10.1016/j.jocn.2018.12.036 30642663 10.1016/j.jocn.2018.12.036

[21] Leu S, Kamenova M, Mariani L, Soleman J (2020) Ultrasound-guided insertion of the ventricular catheter in ventriculoperitoneal shunt surgery: evaluation of accuracy and feasibility in a prospective cohort. Journal of Neurological Surgery Part A: Central European Neurosurgery. 10.1055/s-0040-1714388 10.1055/s-0040-1714388 32968996

[22] Li Y, Chen X, Wang N et al (2019) A wearable mixed-reality holographic computer for guiding external ventricular drain insertion at the bedside. J Neurosurg. 10.3171/2018.4.JNS18124 30485188 10.3171/2018.4.JNS18124

[23] Lund AM (2001) Measuring usability with the USE questionnaire. Usability and User Experience Newsletter, STC Usability SIG, 8(2):3–6.

[24] Mansoor N, Madsbu MA, Mansoor NM et al (2020) Accuracy and complication rates of external ventricular drain placement with twist drill and bolt system versus standard trephine and tunnelation: a retrospective population-based study. Acta Neurochir (Wien). 10.1007/s00701-020-04247-3 32020298 10.1007/s00701-020-04247-3 PMC7066093

[25] Maurer CR Jr, McCrory JJ, Fitzpatrick JM (1993) Estimation of accuracy in localizing externally attached markers in multimodal volume head images. Med Imaging 1993 Image Process. 10.1117/12.154535

[26] Maurer CR Jr, Fitzpatrick JM, Wang MY, Jr RLG, Maciunas RJ, Allen GS (1997) Registration of head volume images using implantable fiducial markers. Med Imaging 1997 Image Process. 10.1117/12.274143 10.1109/42.611354 9263002

[27] Miller C, Tummala RP (2017) Risk factors for hemorrhage associated with external ventricular drain placement and removal. J Neurosurg. 10.3171/2015.12.JNS152341 27035168 10.3171/2015.12.JNS152341

[28] Mofatteh M, Mashayekhi MS, Arfaie S et al (2023) Augmented and virtual reality usage in awake craniotomy: a systematic review. Neurosurg Rev. 10.1007/s10143-022-01929-7 36529827 10.1007/s10143-022-01929-7 PMC9760592

[29] Muralidharan R (2015) External ventricular drains: management and complications. Surg Neurol Int. 10.4103/2152-7806.157620 26069848 10.4103/2152-7806.157620 PMC4450504

[30] Nasa-Task Load Index (NASA-TLX); 20 Years Later - Sandra G. Hart, 2006. https://journals.sagepub.com/doi/10.1177/154193120605000909. Accessed 4 May 2023

[31] Nawabi NLA, Stopa BM, Lassarén P, Bain PA, Mekary RA, Gormley WB (2023) External ventricular drains and risk of freehand placement: a systematic review and meta-analysis. Clin Neurol Neurosurg. 10.1016/j.clineuro.2023.107852 37399698 10.1016/j.clineuro.2023.107852

[32] Ragnhildstveit A, Li C, Zimmerman MH et al (2023) Intra-operative applications of augmented reality in glioma surgery: a systematic review. Front Surg. 10.3389/fsurg.2023.1245851 37671031 10.3389/fsurg.2023.1245851 PMC10476869

[33] Roach J, Gaastra B, Bulters D, Shtaya A (2019) Safety, accuracy, and cost effectiveness of bedside bolt external ventricular drains (EVDs) in comparison with tunneled EVDs inserted in theaters. World Neurosurg. 10.1016/j.wneu.2019.01.106 30735879 10.1016/j.wneu.2019.01.106

[34] Schneider M, Kunz C, Pal’a A, Wirtz CR, Mathis-Ullrich F, Hlaváč M (2021) Augmented reality-assisted ventriculostomy. Neurosurg Focus. 10.3171/2020.10.FOCUS20779 33386016 10.3171/2020.10.FOCUS20779

[35] Schneider M, Kunz C, Wirtz CR, Mathis-Ullrich F, Pala A, Hlavac M (2023) Augmented reality-assisted versus freehand ventriculostomy in a head model. Journal of Neurological Surgery Part A: Central European Neurosurgery. 10.1055/s-0042-1759827 10.1055/s-0042-1759827 37402395

[36] Sharma N, Head JR, Mallela AN et al (2024) Single institution series describing external ventricular drain (EVD) placement and short- and long-term complications related to placement accuracy. Surg Neurol Int. 10.25259/SNI_894_2023 38468651 10.25259/SNI_894_2023 PMC10927197

[37] Stuart MJ, Antony J, Withers TK, Ng W (2021) Systematic review and meta-analysis of external ventricular drain placement accuracy and narrative review of guidance devices. J Clin Neurosci. 10.1016/j.jocn.2021.10.014 34863429 10.1016/j.jocn.2021.10.014

[38] Thavarajasingam SG, Vardanyan R, Arjomandi Rad A et al (2022) The use of augmented reality in transsphenoidal surgery: a systematic review. Br J Neurosurg. 10.1080/02688697.2022.2057435 35393900 10.1080/02688697.2022.2057435

[39] Thomson BR, Gürlek F, Buzzi RM et al (2023) Clinical potential of automated convolutional neural network-based hematoma volumetry after aneurysmal subarachnoid hemorrhage. J Stroke Cerebrovasc Dis. 10.1016/j.jstrokecerebrovasdis.2023.107357 37734180 10.1016/j.jstrokecerebrovasdis.2023.107357

[40] Toma AK, Camp S, Watkins LD, Grieve J, Kitchen ND (2009) External ventricular drain insertion accuracy: is there a need for change in practice? Neurosurgery. 10.1227/01.NEU.0000356973.39913.0B 19934980 10.1227/01.NEU.0000356973.39913.0B

[41] Umana GE, Scalia G, Yagmurlu K et al (2021) Multimodal simulation of a novel device for a safe and effective external ventricular drain placement. Front Neurosci. 10.3389/fnins.2021.690705 34194297 10.3389/fnins.2021.690705 PMC8236630

[42] van Doormaal JAM, Fick T, Ali M, Köllen M, van der Kuijp V, van Doormaal TPC (2021) Fully automatic adaptive meshing based segmentation of the ventricular system for augmented reality visualization and navigation. World Neurosurg. 10.1016/j.wneu.2021.07.099 34333157 10.1016/j.wneu.2021.07.099

[43] van Doormaal JAM, Fick T, Boskovic E, Hoving EW, Robe PAJT, Doormaal TPC (2024) Development and validation of a neurosurgical phantom for simulating external ventricular drain placement. 10.21203/rs.3.rs-5065745/v1 10.1007/s10916-024-02133-4 PMC11698783 39751967

[44] Van Gestel F, Frantz T, Vannerom C et al (2021) The effect of augmented reality on the accuracy and learning curve of external ventricular drain placement. Neurosurg Focus. 10.3171/2021.5.FOCUS21215 34333479 10.3171/2021.5.FOCUS21215

[45] Van Gestel F, Frantz T, Buyck F et al (2023) Neuro-oncological augmented reality planning for intracranial tumor resection. Front Neurol. 10.3389/fneur.2023.1104571 36998774 10.3389/fneur.2023.1104571 PMC10043492

[46] Vychopen M, Kropla F, Winkler D, Güresir E, Grunert R, Wach J (2025) Imaginer 2—improving accuracy with augmented reality navigation system during placement of external ventricular drains over Kaufman’s, Keen’s, Kocher’s and Frazier’s point. Front Surg. 10.3389/fsurg.2024.1513899 39906133 10.3389/fsurg.2024.1513899 PMC11790646

